# Redox-Sensitive VDAC: A Possible Function as an Environmental Stress Sensor Revealed by Bioinformatic Analysis

**DOI:** 10.3389/fphys.2021.750627

**Published:** 2021-12-13

**Authors:** Andonis Karachitos, Wojciech Grabiński, Martyna Baranek, Hanna Kmita

**Affiliations:** Department of Bioenergetics, Faculty of Biology, Institute of Molecular Biology and Biotechnology, Adam Mickiewicz University, Poznań, Poland

**Keywords:** VDAC, parasite, cysteine oxidation, redox sensor, environmental stress, spermatozoa

## Abstract

Voltage-dependent anion-selective channel (VDAC) allows the exchange of small metabolites and inorganic ions across the mitochondrial outer membrane. It is involved in complex interactions that regulate mitochondrial and cellular functioning. Many organisms have several VDAC paralogs that play distinct but poorly understood roles in the life and death of cells. It is assumed that such a large diversity of VDAC-encoding genes might cause physiological plasticity to cope with abiotic and biotic stresses known to impact mitochondrial function. Moreover, cysteine residues in mammalian VDAC paralogs may contribute to the reduction–oxidation (redox) sensor function based on disulfide bond formation and elimination, resulting in redox-sensitive VDAC (rsVDAC). Therefore, we analyzed whether rsVDAC is possible when only one VDAC variant is present in mitochondria and whether all VDAC paralogs present in mitochondria could be rsVDAC, using representatives of currently available VDAC amino acid sequences. The obtained results indicate that rsVDAC can occur when only one VDAC variant is present in mitochondria; however, the possibility of all VDAC paralogs in mitochondria being rsVDAC is very low. Moreover, the presence of rsVDAC may correlate with habitat conditions as rsVDAC appears to be prevalent in parasites. Thus, the channel may mediate detection and adaptation to environmental conditions.

## Introduction

Environmental stress of varying severity always exists in any organism’s habitat. Given the diversity of organisms and their habitats, the diversity may be affected by different environmental conditions. The three main types of environments can be broadly distinguished as terrestrial, aquatic, and semi-terrestrial (semi-aquatic). The habitats available within these environments can be characterized by a set of parameters, including light, temperature, pH, atmospheric or hydrostatic pressure, salinity, and oxygen pressure, as well as individual combinations of these parameters (Edery, 2000; Rothschild and Mancinelli, 2001). An environmental factor is determined as stressful based on the organism’s tolerance against it. Abiotic factors such as temperature, radiation, oxygen pressure, and changes in water availability can exert stress *via* disturbances in gas exchange, water management, and nutrient production (Lesser, 2006; Lushchak, 2011; Sokolova et al., 2012; Wang and Komatsu, 2018). Biotic factors, including predators, competitors, and parasites (e.g., Forsman and Martin, 2009) can also be stressful to organisms. In the case of parasites, both the internal and external environments of a parasitic host dictate the outcome of their infection, resistance, susceptibility, and transmission (Prado et al., 2021). Moreover, the internal conditions of parasitic hosts may constitute a greater constraint upon survival than external conditions (Tinsley, 1999).

Unicellular organisms are exposed to stress conditions through their whole surface. Multicellular organisms regulate their response to stress in a more complex manner, but with cell response as the basis of all response types. Many different organisms are known for their resistance strategies to environmental stress, which indicate efficient cellular anti-stress mechanisms. These mechanisms may protect against intracellular oxidative stress imposed by environmental stress conditions, including increased temperature, not optimal oxygen pressure or high salinity (Laksanalamai and Robb, 2004; Wang et al., 2006; Bagnyukova et al., 2007; Sinha et al., 2013). The state of oxidative stress threatens the functioning of whole cells, especially that of the mitochondria. Reactive oxygen species (ROS), formed mainly during cellular respiration performed by mitochondria, are important signaling molecules but also markers of oxidative stress. Their excess is dangerous due to the direct impact of ROS or ROS-mediated regulation on cell structure and function (e.g., Chung, 2017).

The most common anti-oxidative stress cellular strategy involves maintaining ROS homeostasis. Available data indicate that the homeostasis may be provided by voltage-dependent anion-selective channel (VDAC) (e.g., Shoshan-Barmatz et al., 2010, 2020; De Pinto, 2021; Rostovtseva et al., 2021). This relatively simple, monomeric β-barrel channel at the interface between mitochondria and the cytosol is described as a highly conserved protein of the mitochondrial outer membrane, present in nearly all eukaryotic species examined to date (Colombini, 2012). VDAC performs and regulates inorganic ion and metabolite transport between mitochondria and the cytoplasm under both physiological and pathological conditions (Kroemer et al., 2007; Li et al., 2013; Rostovtseva et al., 2021). The contribution of VDAC in ROS homeostasis (Reina et al., 2010; Sanyal et al., 2020) is based on its transport of superoxide anion (Han et al., 2003), its important role in ROS production (Fang and Maldonado, 2018; Heslop et al., 2020), and its role in regulating the amount and activity of anti-oxidative enzymes (Gałgańska et al., 2008).

Voltage-dependent anion-selective channel is the most abundant protein in the mitochondrial outer membrane and has been relatively well studied since its discovery in 1976 (Schein et al., 1976). As summarized by Rostovtseva et al. (2021) in their comprehensive review, besides being a strictly regulated transport pathway between the mitochondrion and cytosol, VDAC also interacts with a numerous of mitochondrial and cytosolic proteins, which makes the channel a key element in and regulator of communications between mitochondria and cytosol. Moreover, VDAC forms homo- and hetero-complexes with additional functional subunits (e.g., Shoshan-Barmatz et al., 2010, 2018). However, the identification of VDAC paralogs indicates the presence of VDAC variants with slight amino acid differences that undoubtedly perform specified but not yet fully explained functions (De Pinto, 2021).

Three VDAC paralogs were identified in vertebrate mitochondria (Sampson et al., 1997; Messina et al., 2012). The presence of VDAC paralogs was also reported in other multicellular organisms such as invertebrates (Sardiello et al., 2003) and plants (Elkeles et al., 1997; Al Bitar et al., 2003; Wandrey et al., 2004; Lee et al., 2009; Wojtkowska et al., 2012; Hemono et al., 2020; Sanyal et al., 2020), as well as in unicellular organisms such as yeasts *Saccharomyces cerevisiae* (Blachly-Dyson et al., 1997; Di Rosa et al., 2021) and *Candida glabrata* (Wojtkowska et al., 2012) and protists *Trypanosoma brucei* (Flinner et al., 2012) and *Cyanophora paradoxa* (Wojtkowska et al., 2012). Thus, it is assumed that VDAC-encoding genes were duplicated independently in different lineages of eukaryotic organisms, several times during their evolution (Sampson et al., 1996; Saccone et al., 2003; Young et al., 2007). The resulting VDAC-encoding gene redundancy might indicate a need to innovate their existing function and a tendency to duplicate genetic material, as observed in invertebrates, plants, and vertebrates (Saccone et al., 2003). Thus, the following question arises: could the trigger factor be oxidative stress imposed by habitat conditions?

Identifying the function of individual VDAC paralogs is currently one of the main topics concerning VDAC research (De Pinto, 2021). One of the most important aspects of the research is the study of post-translational modification of VDAC paralogs by focusing mainly on cysteine residues. The significance of the number of cysteine residues, as well as their localization and oxidation state in individual mammalian VDAC paralogs, have been indicated by the De Pinto research group (Messina et al., 2012; De Pinto et al., 2016; Reina et al., 2020) and verified by other researchers (Okazaki et al., 2015; Karachitos et al., 2016; Queralt-Martín et al., 2020). The presence of ROS could result in the variable oxidation of cysteine residues exposed to the VDAC interior (including the flexible N-terminal region) or present in the connection loops between 19 β-strands forming the channel (Reina et al., 2010; Okazaki et al., 2015). As oxidative modifications of cysteine residues in VDAC proteins are not detected in other proteins of the mitochondrial outer membrane (Reina et al., 2020), it has been speculated that such modifications could have a regulatory function (including channel gating and conductance) as well as mitochondrial ROS buffering capacity (Okazaki et al., 2015; De Pinto, 2021).

Among the modifications occurring in cysteine residues, disulfide bond formation was shown to affect gating properties and conductance of VDAC (Okazaki et al., 2015; De Pinto et al., 2016; Reina et al., 2020). Accordingly, available data on human VDAC paralogs indicate that cysteine residues in the flexible N-terminal region are crucial for this bond formation. The same probably applies to *Drosophila melanogaster* VDAC paralogs (Komarov et al., 2004). Therefore, the presence of cysteine residues in the N-terminus could be a prerequisite for VDAC to serve as a sensor of the reduction–oxidation (redox) state (Queralt-Martín et al., 2020). Thus, the following questions arise: (1) is the sensor function possible when only one VDAC variant is present in mitochondria, and (2) is the sensor function possible for all VDAC paralogs present in mitochondria? To answer these questions, we used currently available VDAC amino acid sequences to analyze the number of cysteine residues and their location, with particular emphasis on the N-terminus. Next, we examined the relationship between the sequences and the studied species’ ecology. The obtained results suggest that the presence of redox-sensitive VDAC (rsVDAC) proteins may be important for adaptation to environmental conditions.

## Materials and Methods

### Construction of the Database

UniProt (The UniProt Consortium, 2021) was used to compile a list of VDAC paralogs of non-vertebrate and non-plant organisms (Supplementary File 1). Next, the database was enriched with records of paralogs from previously obtained organisms (using sequences of 250–380 amino acids) and with organisms from systematic groups missing in the list. Sequences containing “Fragment” annotations (except of *Hydra vulgaris*) and those whose amino acid sequences did not start with methionine were removed. The list was supplemented with the VDAC sequence predicted for the tardigrade *Milnesium tardigradum*, based on data kindly provided by Felix Bemm (Max Planck Institute for Developmental Biology, Tübingen, Germany). Finally, sequences were blasted using Blastp to determine whether different records for a given organism were paralogs or products of the same genes (Altschul et al., 1997).

### Prediction of the Voltage-Dependent Anion-Selective Channel Structure

The SSPRo (Pollastri et al., 2002; Cheng et al., 2005) and DIpro (Baldi et al., 2004; Cheng et al., 2006) servers available at the Scratch Protein Predictor^1^ were used to estimate the secondary structure of the available VDAC sequences and predict the presence of disulfide bonds, respectively. 3D structures were predicted by applying the Iterative Threading ASSEmbly Refinement (I-TASSER) method (Roy et al., 2010; Yang et al., 2015; Yang and Zhang, 2015). The predicted solutions were visualized using YASARA.^2^

## Results and Discussion

**FIGURE 1:**
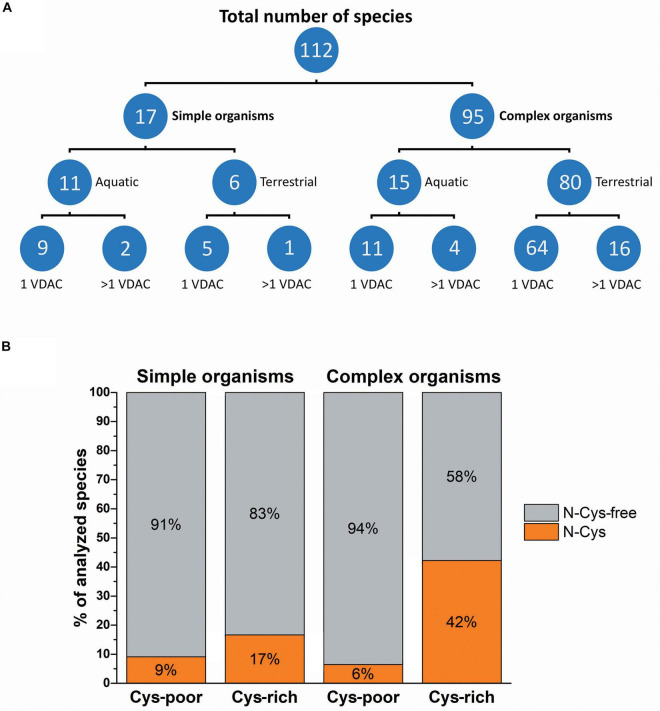
The complexity of organisms and their inhabited environment in relation to the number of VDAC variants and their cysteine residue content. **(A)** The number of organisms classified in terms of complexity, inhabited environment, and the number of genes encoding VDAC proteins. **(B)** Classification of organisms based on the number and localization of cysteine residues in VDAC proteins. Cys-poor: a set of organisms in which only one VDAC variant or at least one VDAC paralog (if present) contains fewer than two cysteine residues in the primary structure; Cys-rich: a set of organisms in which only one VDAC variant or all VDAC paralogs (if present) have more than one cysteine residue in the primary structure; N-Cys: a set of organisms in which only one VDAC variant or all VDAC paralogs (if present) have at least one N-terminal cysteine residue; N-Cys-free: a set of organisms that have at least one VDAC variant with no N-terminal cysteine residue.

To perform all analysis, a database (Supplementary File 1) was built using a collection of species categorized by various parameters, such as a type of environment (terrestrial or aquatic), organism complexity (simple or complex), and the number of VDAC paralogs, including the number (poor or rich) and location (N-terminus free) of cysteine residues (Figure 1). We assumed that a complex organism contained multiple organ systems with different functions (Novikoff, 1945). As shown in Figure 1A, most of the studied organisms categorized by complexity and inhabited environment possess only one VDAC gene. This group mostly includes terrestrial and complex organisms. Due to the lack of VDAC paralogs, various aspects of mitochondrial functioning in these organisms depend on the properties of only one VDAC variant. Therefore, we checked the number of cysteine residues and their location in the secondary structure of VDAC proteins of simple and complex organisms (Figure 1B). We applied the following parameters to perform the operations: one VDAC or at least one VDAC paralog (if present) contained fewer than two cysteine residues in the primary structure (Cys-poor) and one VDAC or all VDAC paralogs (if present) had more than one cysteine residue in the primary structure (Cys-rich). Moreover, the presence of cysteine residue(s) in the N-terminal region upstream of the β1 strand was applied as an additional parameter, as the residue is obligatory for the possibility of disulfide bond formation in human VDAC paralogs (Okazaki et al., 2015; De Pinto et al., 2016; Reina et al., 2020). The results allowed us to distinguish organisms with VDAC(s), in which disulfide bonds could potentially form. Moreover, the possibility of this type of VDAC occurring was more frequent in complex organisms (42%) than in simple organisms (17%).

**FIGURE 2:**
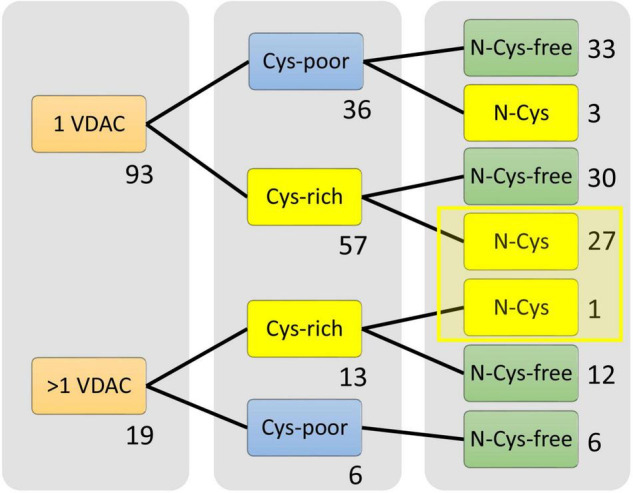
Selection of organisms that possess the only redox-sensitive VDAC (rsVDAC) variant in mitochondria. The analysis indicates the number of cysteine residues and their location in the VDAC secondary structure, as well as the absence or presence of VDAC paralogs, that is, the presence of only one VDAC variant (1 VDAC) or VDAC paralogs (>1 VDAC). Cys-poor: a set of organisms in which only one VDAC variant or at least one VDAC paralog (if present) has fewer than two cysteine residues in the primary structure; Cys-rich: a set of organisms in which only one VDAC variant or all VDAC paralogs (if present) have more than one cysteine residue in the primary structure; N- Cys-free: a set of organisms that have at least one VDAC variant with no N-terminal cysteine residue; rsVDAC, redox-sensitive VDAC.

**FIGURE 3:**
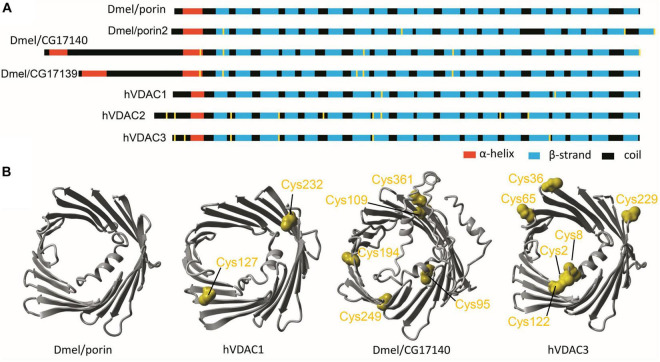
*Drosophila melanogaster* and human VDAC paralogs as models in studies of redox-sensitive VDAC. **(A)** The secondary structure of *D. melanogaster* and human VDAC paralogs with cysteine residues marked in yellow. **(B)** The tertiary structure predicted for ubiquitously expressed *D. melanogaster* and human paralogs (Dmel/porin and hVDAC1, respectively) as well as for paralogs with limited expression, mainly in *D. melanogaster* male reproductive tracts and human testes (Dmel/CG17140 and hVDAC3, respectively). Cysteine residues are marked in yellow.

Next, we analyzed the interactions between the number of cysteine residues, their location in the secondary structure, and the number of VDAC paralogs present in the mitochondria of studied organisms (Figure 2). We found organisms that exclusively contained VDAC characterized by a few cysteine residues (including those within the N-terminus). We assumed that the number and distribution of cysteine residues allowed for their over-oxidation and, consequently, modulation of the channel under oxidative conditions. Therefore, we described this type of VDAC as a rsVDAC. This term refers to data on widely studied VDAC paralogs of humans and *D. melanogaster*. The latter has four VDAC paralogs: Dmel/porin, Dmel/porin2, Dmel/CG17140, and Dmel/CG17139. Dmel/porin is ubiquitous, while the remaining three paralogs are expressed exclusively in the male reproductive organ (Graham and Craigen, 2005). The knockout of *D. melanogaster* VDAC-encoding genes results in partial lethality, mitochondrial respiration defects, abnormal muscle mitochondrial morphology, synaptic dysfunction, and male infertility (Graham et al., 2010). All four paralogs were expressed in yeast *Saccharomyces cerevisiae* cells lacking yVDAC1 (Komarov et al., 2004), and only Dmel/porin and Dmel/porin2 complemented the absence of yVDAC1. Interestingly, the other two paralogs (Dmel/CG17140 and Dmel/CG17139) are characterized by the presence of several cysteine residues, one of which is located within their N-terminus (Figure 3A). Moreover, electrophysiological analysis showed that Dmel/CG17139 does not form a channel. Conversely, Dmel/CG17140 forms a channel in lipid membranes, but is far less voltage-dependent, unlike the canonical Dmel/porin or Dmel/porin2 (Komarov et al., 2004). Thus, Dmel/CG17140 only starts to close slightly at very high potential values (above 110 mV), whereas Dmel/porin2 closes at a potential of approximately 30 mV. Three paralogs, namely VDAC1, VDAC2, and VDAC3, have been detected in humans and other vertebrates. Human VDAC1 (hVDAC1) is ubiquitous and show the highest expression level, whereas hVDAC2 and hVDAC3 are highly abundant in the testes (Yamamoto et al., 2006). Accordingly, VDAC3 knockout in mice causes male infertility (Sampson et al., 2001). Although hVDAC1 and hVDAC2 can rescue the conditional lethal phenotype of yeast cells deficient for yVDAC1, hVDAC3 is almost unable to restore the wild-type phenotype (De Pinto et al., 2010) when the disulfide bond between cysteine residue 2 or 8 (Cys2/Cys8), located at the N-terminus region, and Cys122 is formed (Okazaki et al., 2015). The permanently reduced state of a cluster of close cysteine residues in hVDAC2 and hVDAC3 has been shown to sustain disulfide bond formation in the protein (Pittalà et al., 2020). Such a modification alters the electrophysiological properties of the formed channel, resulting in a lack of voltage dependence of the channel and consequently, the channel remains open. Interestingly, swapping the N-terminus of hVDAC1 with hVDAC3 (which eliminates the N-terminal cysteine residues in hVDAC3) restores the canonical activity of the formed channel and the ability to complement the lack of yVDAC1, as well as confers resistance to yeast against oxidative stress conditions (Reina et al., 2010).

Based on these correlating data, we assume that hVDAC3 and Dmel/CG17140 are orthologs due to their electrophysiological properties and tissue specificity. Both proteins were also used as our model of the assumed rsVDAC (Figure 3B), that is, VDAC containing multiple cysteine residues, with at least one within the N-terminus. The N-terminus is described as the most flexible segment of VDAC, which, in turn, facilitates interactions with other cysteine residues under oxidative conditions (Okazaki et al., 2015). However, it remains unclear if Dmel/CG17139 could be described as an rsVDAC. Based on the present analysis, the secondary structure and distribution of cysteine residues are very similar to Dmel/CG17140; however, limited available experimental data exclude the paralog channel activity.

**FIGURE 4:**
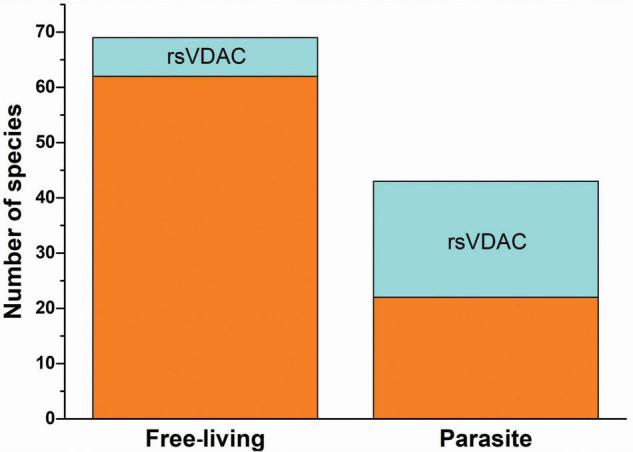
The occurrence of redox-sensitive VDAC in free-living organisms and parasites.

Numerous parasitic species were found in the group of organisms that contained the assumed rsVDAC. As shown in Figure 4, 28 out of 112 studied species were assigned to the rsVDAC group, of which 21 were parasitic species, including obligatory ones (both internal and external) (Table 1). This finding suggests that metabolic and environmental conditions typical for parasitic organisms may support the presence of only one VDAC variant, which may be a redox sensor. In the case of free-living organisms, we noted the presence of at least one VDAC variant that displayed exceptionally low or no probability of cysteine residue oxidation, excluding the function of the redox sensor. Based on the human and *D. melanogaster* models, we believe this type of VDAC to be the most abundant and ubiquitous.

**TABLE 1:** List of organisms with redox-sensitive VDAC being the only VDAC variant, classified by their lifestyle and the presence or absence of cysteine residues in selected regions of the VDAC.

Species	Lifestyle	β1Cys	(β7–β8)Cys	β15Cys	C-Cys
*Steinernema glaseri*	Parasitic (internal)	No	No	No	No
*Wuchereria bancrofti*	Parasitic (internal)	Yes	Yes	Yes	Yes
*Brugia pahangi*	Parasitic (internal)	Yes	No	Yes	Yes
*Loa*	Parasitic (internal)	Yes	Yes	Yes	Yes
*Onchocerca flexuosa*	Parasitic (internal)	Yes	Yes	Yes	Yes
*Litomosoides sigmodontis*	Parasitic (internal)	Yes	No	No	Yes
*Angiostrongylus costaricensis*	Parasitic (internal)	No	No	Yes	No
*Brugia malayi*	Parasitic (internal)	Yes	Yes	Yes	Yes
*Intoshia linei*	Parasitic (internal)	No	No	No	No
*Onchocerca volvulus*	Parasitic (internal)	Yes	Yes	Yes	Yes
*Steinernema carpocapsae*	Parasitic (internal)	Yes	Yes	No	No
*Brugia timori*	Parasitic (internal)	Yes	Yes	Yes	Yes
*Enterobius vermicularis*	Parasitic (internal)	No	No	Yes	No
*Ixodes scapularis*	Parasitic (external)	Yes	No	Yes	No
*Frankliniella occidentalis*	Parasitic (external)	No	No	Yes	No
*Amblyomma aureolatum*	Parasitic (external)	Yes	No	Yes	No
*Rhipicephalus pulchellus*	Parasitic (external)	Yes	No	Yes	No
*Haemaphysalis longicornis*	Parasitic (external)	Yes	No	Yes	No
*Rhipicephalus appendiculatus*	Parasitic (external)	Yes	No	Yes	No
*Ornithodoros turicata*	Parasitic (external)	Yes	No	Yes	No
*Ixodes ricinus*	Parasitic (external)	Yes	No	Yes	No
*Chilo suppressalis*	Free-living	No	No	No	No
*Papilio machaon*	Free-living	No	No	No	No
*Dinothrombium tinctorium*	Free-living	No	No	Yes	No
*Hadrurus spadix*	Free-living	Yes	Yes	Yes	No
*Salpingoeca rosetta*	Free-living	No	No	No	No
*Leptidea sinapis*	Free-living	No	No	No	No
*Operophtera brumata*	Free-living	No	No	No	No

Marine eukaryotic organisms use redox-based mechanisms that mediate sensing and adaptation to environmental stress (Van Creveld et al., 2015). However, little is known about the role of ROS in the signaling of environmental stress conditions. ROS are toxic molecules that can cause severe damage to cells and, therefore, are strictly regulated by a wide range of antioxidant systems (Antonucci et al., 2021; Čapek and Roušar, 2021). However, it is crucial that a moderate amount of ROS act as second messenger molecules in a very complex network of signals in the cell (Tauffenberger and Magistretti, 2021). VDAC is involved in changes in the redox states of the cytosol and mitochondria (Gałgańska et al., 2008) and may act as a mitochondrial oxidative marker, participating in ROS signaling (Reina et al., 2016). Therefore, it is also possible that VDAC participates in the sensing of environmental stress conditions. If that is the case, the evolution of the VDAC structure would depend significantly on the inhabited environment.

**FIGURE 5:**
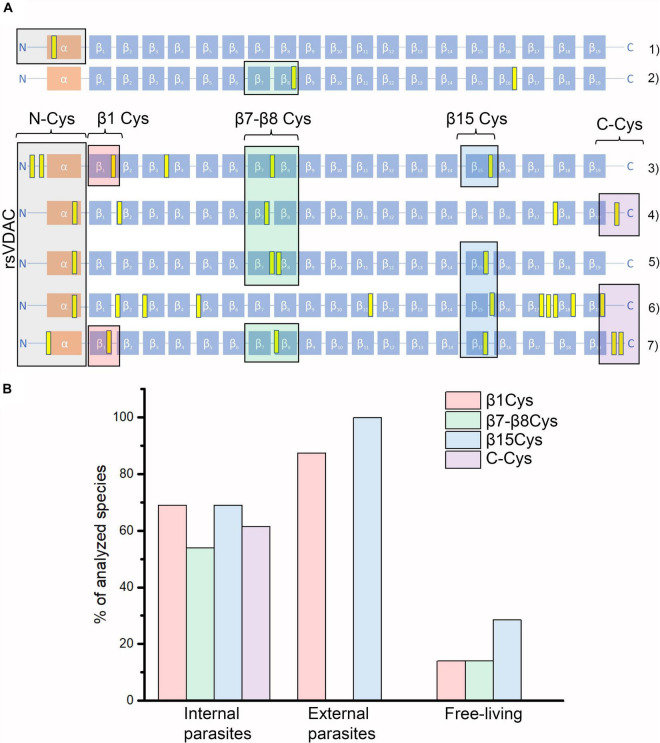
VDAC variants that differ in their number and location of cysteine residues. **(A)** Graphical representations of the studied organisms’ VDAC secondary structure with indicated locations of cysteine residues (marked in yellow). They represent “redox-insensitive” VDAC (1–2) and redox-sensitive VDAC (3–7). (1) *Hypsibius dujardini* (free-living); (2) human VDAC1; (3) human VDAC3; (4) Dmel\CG17140; (5) *Aceria tosichella* (free-living); (6) *Folsomia candida* (free-living); and (7) *Onchocerca flexuosa* (parasitic, internal). **(B)** The percentage of studied organisms with their assumed rsVDAC, being the only VDAC variant in mitochondria, including their lifestyle and the presence of cysteine residues in the selected regions of VDAC proteins (see also Table 1). Besides rsVDAC, *A. tosichella* and *F. candida* possess also “redox-insensitive” VDAC.

*Cyanidioschyzon merolae* is a unicellular extremophilic eukaryotic organism adapted to high-sulfur acidic hot spring habitats. This organism has only one gene encoding VDAC, which contains four cysteine residues, all of which are located outside the N-terminus. By contrast, the tardigrade *Hypsibius dujardini*, which can survive under extreme conditions, also has one VDAC, with the protein containing only one cysteine residue located at the N-terminus. Both the VDAC proteins did not qualify as rsVDAC in our analysis. Instead, the group of organisms with the assumed rsVDAC was dominated by parasites. Using hVDAC3 as the rsVDAC model, we verified the presence and location of cysteine residues in assumed rsVDAC being the only VDAC variant in mitochondria. The regions containing cysteine residues included the N-terminus, the β1 strand, the segment containing β7 and β8 strands and the β15 strand (Figure 5A). Cysteine residues in the β1 and β15 strands turned out to be quite common in parasites, whereas those in the β7 and β8 segment were characteristic of internal parasites (Figure 5B and Table 1). In hVDAC3, the β7 and β8 segment is the location for Cys122, which is responsible for forming disulfide bonds with the N-terminal cysteine residues (Okazaki et al., 2015). A cysteine residue is also present in this region of Dmel/CG17140 (Figures 3, 5A), suggesting its potential role in redox sensitivity. Accordingly, the presence of two cysteine residues in modified mouse VDAC1, one at the N-terminus (Val3Cys) and the second one in β7 segment (Lys119Cys) resulted in the formation of disulfide bond and strong deviation from the typical native channel gating under oxidative condition (Mertins et al., 2012). In further studies, it was demonstrated that the N-terminal dynamics were essential for voltage gating (Zachariae et al., 2012; Zeth and Zachariae, 2018). We also indicated the presence of cysteine residue(s) at the C-terminus only in internal parasites (Table 1). It should be noted that cysteine residue(s) in the location is (or are) less common in VDAC of free-living organisms that have only one VDAC variant meeting our criteria of rsVDAC.

Thus, what are the features that distinguish parasites from free-living organisms? It is suggested that hosts of parasites can be considered a safe environment, and the external environment to which parasites are exposed, for example, during transmission, as hostile ones (Tinsley, 2007). Conversely, many years of coexistence with the host body requires suitable adaptation, such as a strong antioxidant system, which may serve as a defense strategy against host-generated ROS (Chiumiento and Bruschi, 2009). Perhaps the reduced imbalance between ROS generated by the host and the antioxidant system requires the presence of stronger redox sensors in some parasites. The same idea may apply to mature spermatozoa enriched in rsVDAC (Reina et al., 2016). Spermatozoa are foreign to both the male who produces them and the female who receives them (Clark and Schust, 2013). The organs of the female reproductive tract are subject to being colonized by pathogens and, therefore, have developed multiple adaptations to impede the invasion and proliferation of such pathogens. In addition to physical (production of a cleansing outward flow of fluid and secretion of a viscoelastic mucus) and chemical (acidification of the vaginal fluid) impediments in the female tract, immunological barriers could include components of the innate immune system, including inflammatory responses, ROS, and antimicrobial peptides, that could potentially damage spermatozoa (Ford, 2004; Wigby et al., 2019).

## Conclusion

The imbalance between ROS production and antioxidant capacity – which causes oxidative stress – is a common feature of cells exposed to environmental stress conditions. Our hypothesis was based on the assumption that, in some organisms, VDAC amino acid sequences form proteins sensitive to redox changes. Specifically, cysteine residues can potentially be oxidized and form disulfide bonds that alter the properties of the formed channel. The available data indicate that the bond formation requires the presence of cysteine residue in the flexible N-terminus. A large group of organisms, most often possessing only one VDAC variant, do not contain this type of VDAC that we termed “redox-sensitive.” Redox-sensitive VDACs may form adjacent to “redox-insensitive” ones in organisms with VDAC paralogs, such as *D. melanogaster* or humans. The redox-sensitive paralogs are often expressed in specific tissues and are not ubiquitous. Finally, our observations indicate that certain organisms – mainly parasites – have only one, but potentially redox-sensitive, VDAC variant. Thus, there is a possibility that VDAC evolution may depend on environmental conditions and that the channel may mediate detection and adaptation to environmental stress.

## Data Availability Statement

The original contributions presented in the study are included in the article/Supplementary Material, further inquiries can be directed to the corresponding author.

## Author Contributions

AK conceived, designed, and made the analyses. HK supervised the performed analyses. AK and HK wrote the final version of the manuscript. MB contributed to the database and wrote sections of the manuscript. WG created the database, helped in the analysis, and wrote sections of the manuscript. All authors have read and approved the final manuscript.

## Conflict of Interest

The authors declare that the research was conducted in the absence of any commercial or financial relationships that could be construed as a potential conflict of interest.

## Publisher’s Note

All claims expressed in this article are solely those of the authors and do not necessarily represent those of their affiliated organizations, or those of the publisher, the editors and the reviewers. Any product that may be evaluated in this article, or claim that may be made by its manufacturer, is not guaranteed or endorsed by the publisher.

## Footnotes

1
http://scratch.proteomics.ics.uci.edu/

2
www.yasara.org
